# Diagnostic value of multi-gene methylation in colorectal cancer screening

**DOI:** 10.3389/fonc.2025.1660035

**Published:** 2026-01-09

**Authors:** Qingyan Yang, Liming Chen

**Affiliations:** 1 Department of Medical Oncology, Eastern Guangdong Hospital of the Third Affiliated Hospital of Sun Yat-sen University, Meizhou, China; 2 Shantou University Medical College, Shantou, China; 3 Department of Oncology, The First Affiliated Hospital of Shantou University Medical College, Shantou, China

**Keywords:** colorectal cancer, multi-gene, methylation, screening, diagnostic value

## Abstract

**Objective:**

To evaluate the diagnostic value and clinical application of multi-gene methylation assay in colorectal cancer (CRC) screening.

**Methods:**

This was a single-center, retrospective, real-world study conducted at the First Affiliated Hospital of Shantou University Medical College, China. A total of 450 participants were enrolled from January 2023 to December 2024, including 150 healthy individuals, 120 colorectal polyp patients, and 180 CRC patients. The methylation status of six plasma-derived loci (SEPT9-R1, SEPT9-R2, BCAT1, IKZF1, BCAN, and VAV3) was assessed using real-time multiplex quantitative PCR (qPCR). The diagnostic performance and predictive factors of the multi-gene methylation assay for CRC detection were assessed.

**Results:**

The multi-gene methylation assay effectively differentiated healthy controls, colorectal polyp patients, and CRC patients (P < 0.001), and methylation levels increased with advancing tumor stage. The combined panel demonstrated superior diagnostic accuracy over single-gene assays, yielding an AUC of 0.958 (95% CI: 0.934–0.982) in the training cohort and 0.952 (95% CI: 0.920–0.984) in the validation cohort. Multivariate analysis further identified tumor size (OR = 1.974, 95% CI: 1.321–2.950, P = 0.005) and TNM stage (OR = 2.117, 95% CI: 1.452–3.087, P = 0.002) as independent predictors of multi-gene methylation positivity.

**Conclusion:**

Multi-gene methylation detection showed high diagnostic value for CRC and may serve as a valuable adjunct tool in CRC screening strategies.

## 1 Introduction

Colorectal cancer (CRC) is one of the most common malignancies worldwide and remains a major cause of cancer-related morbidity and mortality (1, 2). Early detection is essential for improving patient outcomes (3). Although fecal occult blood testing and colonoscopy are widely used, their clinical application is limited by invasiveness, patient compliance, and resource requirements, making population-based screening challenging (4).

DNA methylation alterations occur early in CRC development and serve as promising biomarkers for early detection (5, 6). Circulating cell-free DNA (cfDNA) methylation testing offers a minimally invasive alternative with improved patient acceptance (7). The SEPT9 methylation assay has been approved by the U.S. FDA, underscoring the value of methylation-based screening (8). However, single-gene detection has shown limited sensitivity and specificity (9).

To improve diagnostic accuracy, Cai et al. developed ColonAiQ, a six-gene cfDNA methylation panel (SEPT9-R1, SEPT9-R2, BCAT1, IKZF1, BCAN, and VAV3). These markers were identified through high-throughput screening and supported by literature demonstrating aberrant methylation and biological relevance in CRC development (10). A subsequent multicenter prospective study in China validated the panel’s ability to detect recurrence and support risk stratification (11). However, evidence on its performance in routine clinical practice remains limited. Therefore, we conducted a single-center real-world study to evaluate its clinical applicability and provide additional evidence for its utility in early CRC screening.

## 2 Materials and methods

### 2.1 Study design

This was a single-center, retrospective, real-world study conducted at the First Affiliated Hospital of Shantou University Medical College between January 2023 and December 2024. Eligible participants included patients diagnosed with CRC, patients with colorectal polyps, and healthy individuals undergoing routine physical examinations. Participants were recruited from the Departments of Gastroenterology, Oncology, and Gastrointestinal Surgery (both outpatient and inpatient), as well as from the health examination center. The study was approved by the Ethics Committee of the First Affiliated Hospital of Shantou University (approval No. II2025-048-01). Informed consent was not required as the study was retrospective, and data collected was de-identified.

### 2.2 Study population

Inclusion criteria: (1) CRC group: Patients aged 18–75 years with histologically confirmed CRC (via colonoscopic biopsy or surgical pathology), who had not received prior antitumor treatments (e.g., chemotherapy or radiotherapy), and with complete clinical data including imaging and laboratory results (12). (2) Polyp group: Patients aged 18–75 years with colorectal polyps detected by colonoscopy, histologically confirmed as adenomatous polyps, with a polyp diameter ≥5 mm and complete clinical data (13). (3) Healthy group: Individuals aged 18–75 years with normal results on routine physical examination, including blood tests, biochemistry, tumor markers, abdominal ultrasound, and chest CT; no history of malignancy in the past five years; no polyps or other lesions detected by colonoscopy; and complete clinical data.

Exclusion criteria: (1) Other malignancies; (2) severe cardiac, hepatic, or renal dysfunction; (3) autoimmune diseases such as systemic lupus erythematosus or rheumatoid arthritis; (4) acute infectious diseases (bacterial or viral); (5) pregnant or lactating women; (6) psychiatric disorders affecting compliance; (7) inflammatory bowel disease; (8) familial adenomatous polyposis or hereditary nonpolyposis colorectal cancer.

### 2.3 Sample collection and processing

Peripheral venous blood (10 mL) was collected in EDTA-K2 tubes from fasting participants in the morning and gently inverted 8–10 times. Samples were processed within 2 hours. Plasma was obtained by centrifugation at 3,000 × g for 10 minutes and aliquoted (≥ 4 mL total; 0.5 mL per tube) into RNase-free tubes, then stored at −80°C to avoid repeated freeze–thaw cycles.

Before cfDNA extraction, plasma was thawed at room temperature. cfDNA was isolated using a magnetic bead-based protocol per the manufacturer’s instructions. Approximately 20 ng cfDNA (minimum ≥10 ng) was used for bisulfite conversion, and DNA was eluted in 15 µL. Purity was assessed by NanoDrop (A260/280 of 1.8–2.0). All cfDNA extraction and methylation analyses were performed at Guangzhou Huayin Medical Laboratory.

### 2.4 cfDNA methylation detection

cfDNA was isolated from plasma and subjected to bisulfite conversion according to the manufacturer’s instructions. Converted DNA was used immediately or stored at 2–8°C for up to 24 h. Plasma ctDNA methylation was quantified using the ColonAiQ multi-gene methylation assay (Singlera Genomics, Shanghai, China), a commercial qPCR-based platform for colorectal cancer detection. The assay targets six CRC-associated methylation markers (SEPT9-R1, SEPT9-R2, BCAT1, IKZF1, BCAN, and VAV3).

Methylation levels were measured by multiplex real-time PCR, and ΔCt values for each marker were analyzed using a proprietary logistic-regression algorithm to generate individual marker scores. A gene was considered methylation-positive if P ≥ 6.0. For the multi-gene panel, a sample was classified as methylation-positive if at least one of the six genes was positive. All reactions were performed in triplicate and incorporated into the algorithm rather than interpreted by a single Ct threshold. The qPCR procedures and reporting follow the MIQE guidelines (14, 15). Details of the method are available in previous published study (10).

This algorithm-based strategy has been validated in large clinical cohorts and demonstrated high diagnostic performance for CRC (11). Reporting of diagnostic accuracy in this study adheres to the STARD 2015 guidelines (16). As the platform is proprietary, primer and probe sequences are not publicly disclosed; however, assay reproducibility and methodological transparency are supported by publicly available documentation and peer-reviewed validation studies (10).

### 2.5 Clinical data collection

Clinical information was extracted from the hospital’s electronic medical record system. Collected data included sex, age, clinical symptoms, Family History of CRC, CT/MRI findings, tumor location, cancer staging and laboratory test indicators.

### 2.6 Sample size

Based on previously reported data, the positive rate of the multi-gene methylation assay in CRC was assumed to be approximately 85%, whereas the corresponding rate in patients with polyps or other non-CRC conditions was estimated to be around 60% (10). Using a two-sided test with a significance level of 0.05, a statistical power of 0.90, and an equal allocation ratio (1:1), the required sample size was calculated with PASS 2021 (two-independent-proportions procedure), yielding a minimum of 130 subjects in total (65 per group).

### 2.7 Statistical analysis

Statistical analyses were performed using SPSS version 25.0. Continuous variables were expressed as mean ± standard deviation ( $\bar{\text{x}}$ ± s) and compared using independent sample t-tests or one-way ANOVA. Categorical variables were reported as counts and percentages, and comparisons were made using the χ² test or Fisher’s exact test.

Participants were randomly stratified into training and validation sets at a 7:3 ratio. Propensity score matching (PSM) was applied for pairwise comparisons (healthy vs. polyp, polyp vs. CRC) at a 1:1 ratio based on matching variables such as age and sex. An ordinal logistic regression model was constructed for multi-gene methylation diagnosis.

The diagnostic performance of individual and combined methylation markers was evaluated using receiver operating characteristic (ROC) curve analysis. Area under the curve (AUC), sensitivity, specificity, positive predictive value (PPV), and negative predictive value (NPV) were calculated. Internal validation was performed using the bootstrap method with 2000 resampling iterations.

The Cochran–Armitage trend test was used to assess the increasing trend of methylation positivity across the three-stage disease progression (healthy → polyp → CRC). Multivariate logistic regression models were constructed to explore potential predictive factors associated with multi-gene methylation positivity, with results presented as odds ratios (ORs) and 95% confidence intervals (CIs). A two-sided P-value < 0.05 was considered statistically significant.

## 3 Outcome

### 3.1 Study population

**Figure 1:**
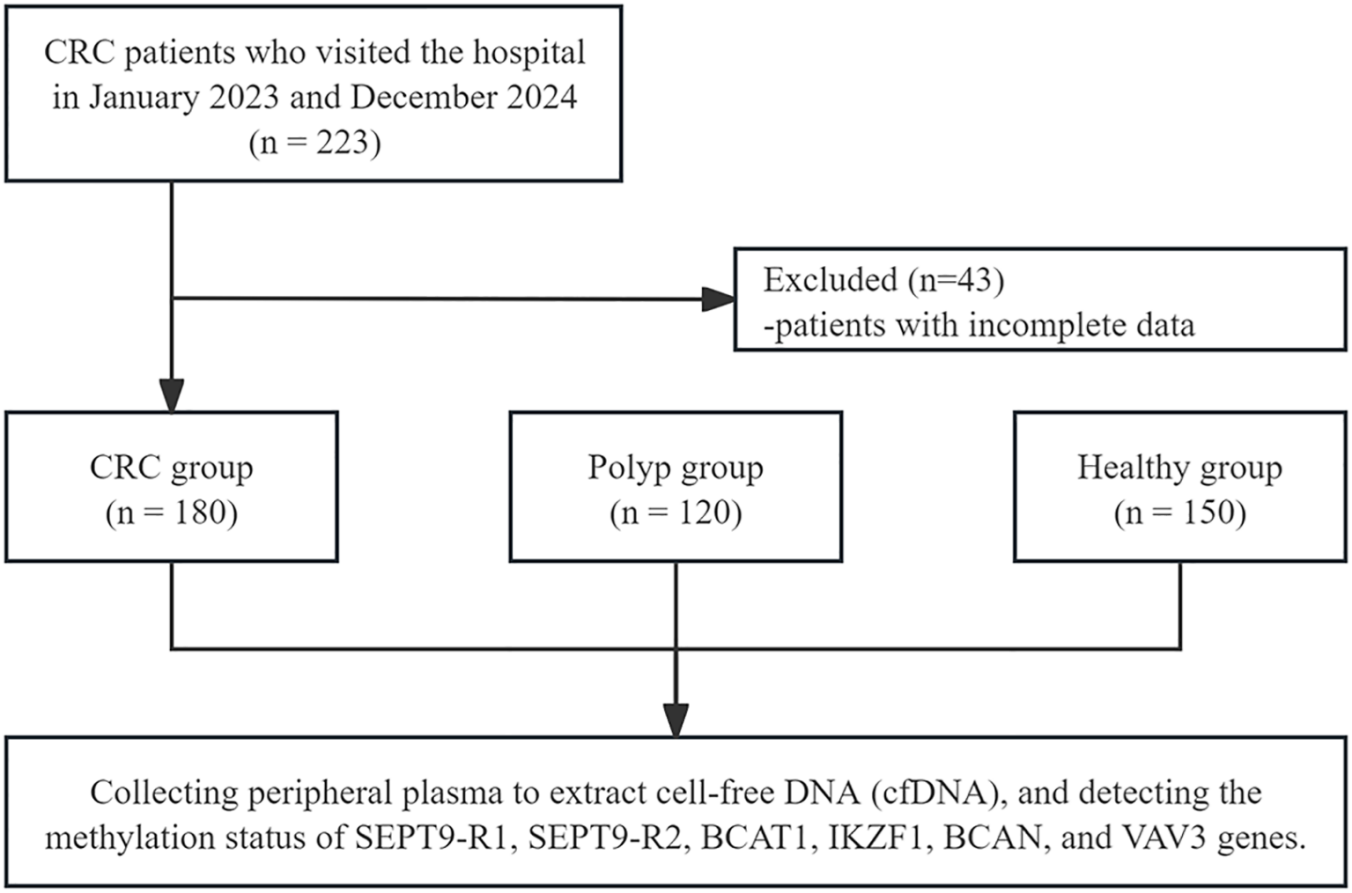
Flow chart illustrating the selection of patients.

A total of 180 CRC patients (CRC group), 120 colorectal polyp patients (polyp group), and 150 healthy individuals (healthy group) were enrolled. In the CRC group, there were 104 males and 76 females, with a mean age of 62.8 ± 8.5 years old. According to the TNM staging system, there were 36 patients in stage I, 52 in stage II, 62 in stage III, and 30 in stage IV. The tumor was located in the colon in 99 cases and in the rectum in 81 cases. In the polyp group, 68 were male and 52 were female, with a mean age of 58.2 ± 9.8 years old. All polyps were histologically confirmed as adenomas, including 72 tubular adenomas, 28 villous adenomas, and 20 tubulovillous adenomas. In the healthy group, there were 82 males and 68 females, with a mean age of 56.5 ± 10.2 years old. No statistically significant differences were found with respect to sex, age, and BMI (P > 0.05), whereas higher levels of CEA and CA19–9 were observed in the CRC group (P < 0.05). The study flowchart is presented in **Figure 1**, and the baseline characteristics of the participants are presented in **Table 1**.

**Table 1:** Baseline characteristics of patients in the healthy group, polyp group, and colorectal cancer group.

Characteristics	CRC group (n = 180)	Polyp group (n = 120)	Healthy group (n = 150)	t/χ2	*P* value
Age, years, mean (SD)	62.8 (8.5)	58.2 (9.8)	56.5 (10.2)	1.403	0.161
Male, n (%)	104 (57.8)	68 (56.7)	82 (54.7)	0.366	0.545
BMI, kg/m^2^, mean (SD)	23.25 (3.75)	22.89 (3.63)	22.90 (3.25)	1.130	0.259
Smokers, n (%)	107 (59.1)	54 (45.4)	70 (46.7)	0.048	0.852
Drinkers, n (%)	96 (53.0)	56 (47.1)	58 (38.7)	2.016	0.166
Family history of CRC, n (%)	35 (19.3)	16 (13.4)	11 (7.3)	7.968	0.005
FIT-positive, n (%)	113 (62.4)	36 (30.3)	33 (22.0)	89.770	< 0.001
HGB, g/L, mean (SD)	87.60 (27.12)	126.35 (26.35)	142.22 (23.45)	10.293	< 0.001
CEA, log_10_μg/mL, mean (SD)	2.43 (0.75)	1.32 (0.51)	1.23 (0.59)	18.753	< 0.001
CA19-9, log_10_IU/mL, mean (SD)	2.46 (0.70)	1.90 (0.38)	1.83 (0.45)	11.246	< 0.001

CRC, colorectal cancer; BMI, Body Mass Index; FIT, Fecal Immunochemical Test; HGB, Hemoglobin; CEA, Carcinoembryonic Antigen; CA19-9, Carbohydrate Antigen 19-9.

### 3.2 Methylation status of multi-genes among groups

The methylation-positive rates of the six gene loci (SEPT9-R1, SEPT9-R2, BCAT1, IKZF1, BCAN, and VAV3) across the healthy, colorectal polyp, and CRC groups are summarized in **Table 2**. All six loci demonstrated significant differences among the three groups (all P < 0.001), with a clear stepwise increase from healthy controls to patients with polyps and then to CRC (all trend P < 0.001). Specifically, the multi-gene positivity rate was lowest in the healthy group (9.3%), intermediate in the polyp group (56.7%), and highest in the CRC group (91.7%).

**Table 2:** Comparison of gene methylation detection results among three groups.

Group	n	SEPT9-R1	SEPT9-R2	BCAT1	IKZF1	BCAN	VAV3	Multi-gene combined
Healthy group, n, (%)	150	8 (5.3)	6 (4.0)	7 (4.7)	5 (3.3)	6 (4.0)	4 (2.7)	14 (9.3)
Polyp group, n, (%)	120	38 (31.7)	35 (29.2)	42 (35.0)	36 (30.0)	34 (28.3)	32 (26.7)	68 (56.7)
CRC group, n, (%)	180	158 (87.8)	152 (84.4)	162 (90.0)	156 (86.7)	148 (82.2)	144 (80.0)	165 (91.7)
χ^2^		238.45	226.82	246.73	239.21	221.46	215.38	252.64
P-value		<0.001	<0.001	<0.001	<0.001	<0.001	<0.001	<0.001
Trend P-value		<0.001	<0.001	<0.001	<0.001	<0.001	<0.001	<0.001

### 3.3 Methylation levels by TNM stage

**Figure 2:**
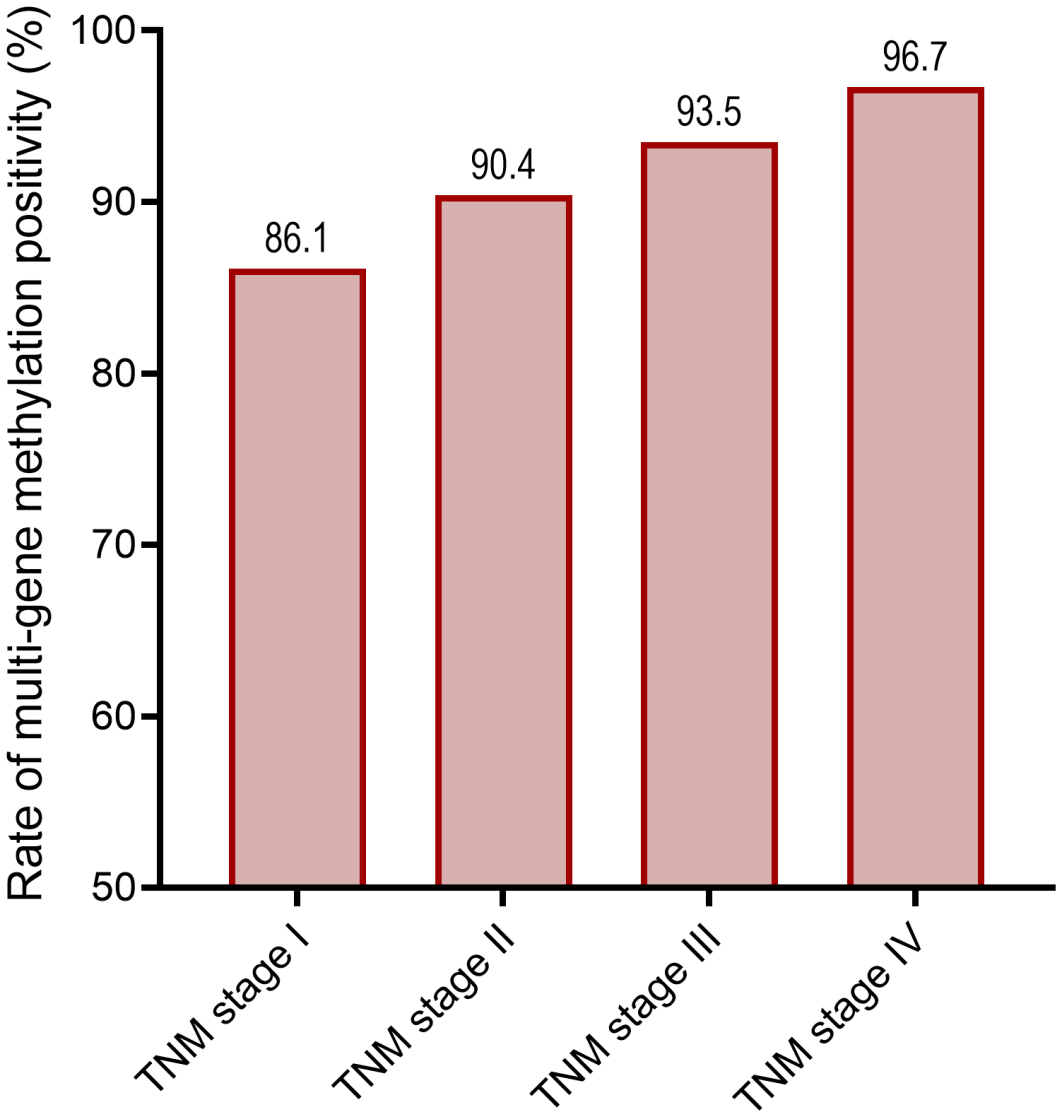
Multi-gene methylation positivity rate across different TNM stages.

The positive rates of multi-gene methylation were further analyzed across different TNM stages of CRC. A progressive increase in methylation positivity was observed with advancing tumor stage, and the differences were statistically significant (P < 0.05 for all markers). Notably, the positivity rate of the multi-gene methylation rose from 86.1% in stage I to 96.7% in stage IV, indicating a strong positive association between methylation burden and tumor severity (**Figure 2**; **Supplementary Table 1**).

### 3.4 Diagnostic performance of the multi-gene methylation assay

**Figure 3:**
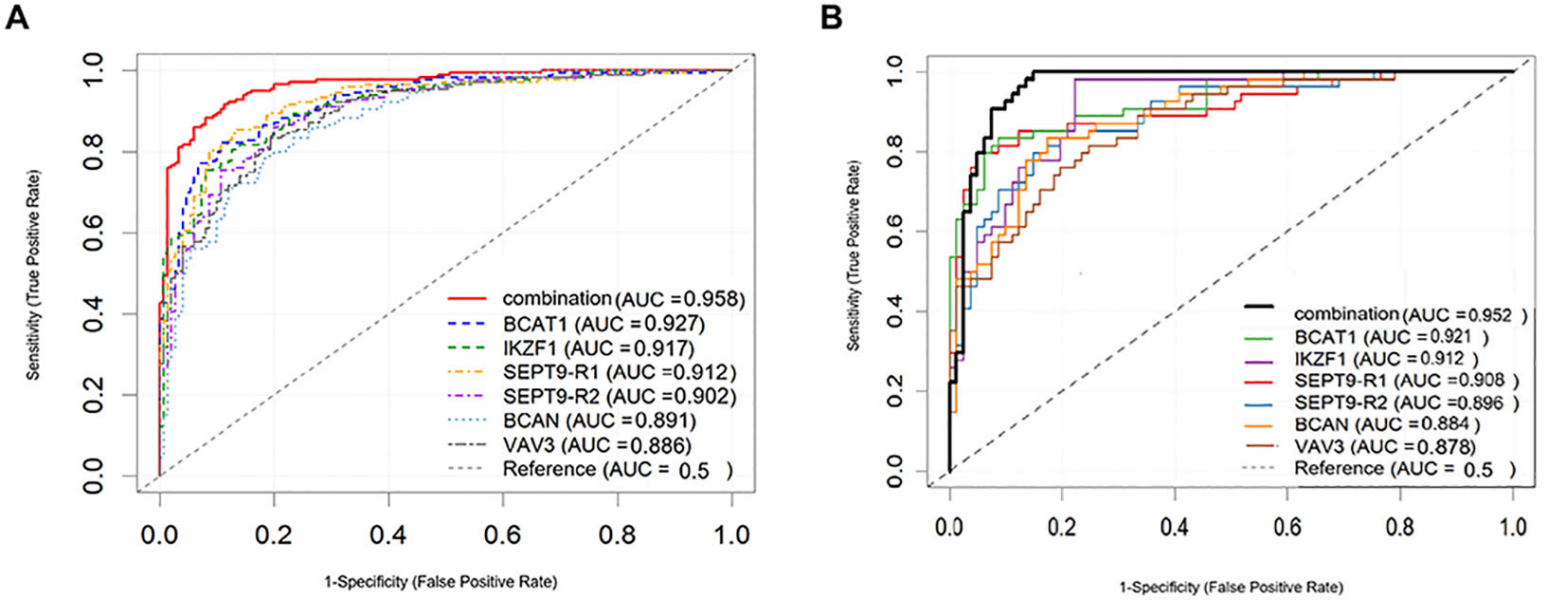
ROC curves of the multi-gene methylation assay in **(A)** the training cohort and **(B)** the validation cohort of CRC patients.

CRC patients were randomly and stratifiedly assigned to a training set and a validation set in a 7:3 ratio. Baseline characteristics for both cohorts are shown in **Supplementary Table 2**, with no significant differences observed between the groups. The multi-gene methylation assay demonstrated excellent overall performance for CRC detection, with a sensitivity of 91.7% and specificity of 93.3%. The AUC were 0.958 (95% CI: 0.934–0.982) in the training cohort and 0.952 (95% CI: 0.920–0.984) in the validation cohort, outperforming any single-gene marker (**Figure 3**). The overall diagnostic accuracy was 92.4%, with a PPV of 94.3% and an NPV of 90.3%. Detailed results are presented in **Table 3**.

**Table 3:** Diagnostic efficacy analysis of each gene methylation detection.

Statistical indicators	SEPT9-R1	SEPT9-R2	BCAT1	IKZF1	BCAN	VAV3	Multi-gene combined
Sensitivity (%)	87.8	84.4	90.0	86.7	82.2	80.0	91.7
Specificity (%)	94.7	96.0	95.3	96.7	96.0	97.3	93.3
AUC (Training cohort)	0.912	0.902	0.927	0.917	0.891	0.886	0.958
95%CI	0.881-0.943	0.870-0.934	0.897-0.957	0.886-0.948	0.857-0.925	0.851-0.921	0.934-0.982
AUC (Validation cohort)	0.908	0.896	0.921	0.912	0.884	0.878	0.952
95%CI	0.868-0.948	0.853-0.939	0.883-0.959	0.871-0.953	0.840-0.928	0.832-0.924	0.920-0.984
Accuracy (%)	91.0	89.8	92.5	91.4	88.7	88.2	92.4
PPV (%)	94.0	94.4	94.7	94.5	93.7	93.5	94.3
NPV (%)	88.2	86.5	90.9	87.9	85.1	83.6	90.3

### 3.5 Clinical predictors of multi-gene methylation in CRC detection

Multivariate analysis was conducted to evaluate the association between clinical characteristics and multi-gene methylation positivity in CRC patients. The results showed that tumor size (OR = 1.974, 95% CI: 1.321–2.950, P = 0.005) and TNM stage (OR = 2.117, 95% CI: 1.452–3.087, P = 0.002) were independent predictors of multi-gene methylation positivity (**Supplementary Table 3**).

## 4 Discussion

Our study evaluated the performance of a multi-gene cfDNA methylation assay for CRC screening in a large real-world cohort, specifically assessing its ability to distinguish CRC from colorectal polyps and healthy individuals. The results demonstrated high diagnostic accuracy, with high sensitivity and specificity. This non-invasive and convenient test offering a promising tool for early CRC screening.

The six genes loci included in the ColonAiQ panel are functionally relevant to CRC development and progression. SEPT9, frequently hypermethylated in CRC, loses tumor-suppressive activity and contributes to malignant transformation. Previous study indicated that the two targeted regions of SEPT9 (R1 and R2) showed positivity rates of 87.8% and 84.4%, with specificities of 94.7% and 96.0%, respectively, among CRC patients (9). BCAT1 promotes tumor growth by supporting amino acid metabolism, redox balance, and oncogenic signaling (17). IKZF1 regulates cell proliferation, DNA repair, and apoptosis, and its dysregulation has been linked to CRC initiation (18). BCAN remodels the extracellular matrix and activates pro-invasive pathways, facilitating tumor invasion (19). VAV3 drives cell migration, proliferation, and survival through Rho/Rac GTPase signaling (20). Aberrant methylation of these genes has been reported in CRC tissues and plasma, providing both mechanistic rationale and clinical support for their inclusion in multi-gene cfDNA assays for early CRC detection and risk stratification (21–25).

However, given that CRC development involves multiple signaling pathways, relying on a single biomarker is often insufficient for accurate diagnosis. Multi-gene methylation assays offer a more comprehensive view of the tumor’s molecular landscape by capturing alterations from various biological pathways (26). Our studies showed that an ordinal logistic regression model incorporating all six genes loci showed better diagnostic performance than any individual gene. The multi-gene combination achieved an AUC of 0.958 in the training cohort and 0.952 in the validation cohort, confirming the robustness and generalizability of the model.

The methods of multi-gene methylation assay using ColonAiQ panal have been validated in previous studies. Cai et al. developed the ColonAiQ assay by systematically screening cancer-specific methylation regions from tissue and plasma samples, ultimately identifying six high-performance markers for CRC detection. In a multicenter validation study, ColonAiQ demonstrated excellent diagnostic accuracy (AUC = 0.93 for CRC), superior early-stage detection compared with FIT, and potential utility for postoperative surveillance (10). Subsequently, a prospective multicenter cohort study in China including 299 patients with stage I–III CRC reported that patients with positive multi-gene methylation status had a 17.5-fold higher risk of recurrence at one month post-surgery compared with ctDNA-negative patients (hazard ratio [HR] = 17.5, 95% CI 8.9-34.4, P<0.001). Moreover, recurrence-free survival was significantly shorter in methylation-positive patients than in ctDNA-negative patients (HR = 13.8, 95% CI 5.9–32.1, P<0.001) (11).

By enrolling 450 participants, we observed a stepwise increase in methylation positivity of multi-gene from healthy individuals to those with polyps and finally to CRC patients. Notably, the combined six-gene panel achieved a methylation positivity rate of 91.7% patients with CRC, compared to 56.7% in the polyp group and 9.3% observed in healthy individuals, highlighting its ability to identify high-risk individuals. On the other hand, our study found a strong correlation between gene methylation levels and TNM stage, with a progressive increase in methylation positivity as the tumor stage advanced. Notably, the multi-gene methylation assay maintained a high positivity rate even in patients with early-stage disease (TNM stage I–II), supporting its applicability and reliability for early CRC screening.

Further analyses showed that methylation positive were significantly linked to TNM stage and tumor size. Tumor progression and increased tumor burden are usually accompanied by higher levels of tumor-related circulating DNA and more widespread epigenetic abnormalities. As tumors grow and advance in stage, more methylated DNA is released into the bloodstream. This makes detection more likely and leads to higher methylation positive rates (27).

This study has several strengths. First, it is a large real-world study to assess the value of SEPT9-R1, SEPT9-R2, BCAT1, IKZF1, BCAN, and VAV3 methylation in CRC screening. We demonstrated that the multi-gene methylation panel provides strong diagnostic performance and supports clinical application. Second, we evaluated its performance across disease stages and found that positivity remained high even in early-stage CRC, underscoring its potential for early detection. Third, we analyzed clinical factors associated with methylation positivity and identified advanced TNM stage and larger tumor size as risk factors. These findings offer practical guidance for clinical interpretation, including incorporating patient risk profiles when evaluating positive results.

However, our study also has limitations. First, it was conducted at a single center in a real-world setting. Although we performed internal validation using training and validation datasets, external validation in independent cohorts is needed to confirm generalizability. Second, the association between clinical features and methylation positivity should be interpreted cautiously due to the limited sample size. Larger prospective studies and mechanistic research are required to validate these findings.

In conclusion, our findings demonstrated that plasma-based multi-gene methylation profiling of cfDNA provides a highly accurate and noninvasive strategy for the detection of CRC and high-risk polyps. Larger tumors and more advanced TNM stages may be associated with higher rate of multi-gene methylation. Future prospective studies with larger sample sizes are needed to further validate its value in early colorectal cancer screening.

## Data Availability

The original contributions presented in the study are included in the article/**Supplementary Material**. Further inquiries can be directed to the corresponding authors.
