# Supplementary material for: Diagnostic value of multi-gene methylation in colorectal cancer screening
Source: Front Oncol. 2026 Jan 9;15:1660035. doi: 10.3389/fonc.2025.1660035 (PMC12827121; doi:10.3389/fonc.2025.1660035)

Supplementary materials

Supplementary Table 1. Gene methylation positivity rates in patients with different TNM stages.

Supplementary Table 2. Baseline data of CRC patients in the training and validation cohorts.

Supplementary Table 3. Clinical variables associated with multi-gene methylation positive in multivariate analysis.

Supplementary Figure 1. Representative amplification curves for the methylated standards.

Supplementary Table 1. Gene methylation positivity rates in patients with different TNM stages.

TNM stage	n	SEPT9-R1	SEPT9-R2	BCAT1	IKZF1	BCAN	VAV3	Six-gene combined
Stage I [n, (%)]	36	28(77.8)	26(72.2)	30(83.3)	28(77.8)	26(72.2)	24(66.7)	31(86.1)
Stage II [n, (%)]	52	44(84.6)	42(80.8)	46(88.5)	44(84.6)	42(80.8)	40(76.9)	47(90.4)
Stage III [n, (%)]	62	56(90.3)	54(87.1)	58(93.5)	56(90.3)	52(83.9)	52(83.9)	58(93.5)
Stage IV	30	30(100.0)	30(100.0)	28(93.3)	28(93.3)	28(93.3)	28(93.3)	29(96.7)
χ^2	-	9.842	10.236	4.628	6.842	7.438	8.926	4.382
P-value	-	0.020	0.017	0.201	0.077	0.059	0.030	0.223
Trend P-value	-	0.003	0.002	0.134	0.026	0.018	0.007	0.086

Supplementary Table 2. Baseline data of CRC patients in the training and validation cohorts.

	**Training cohort (n=126)**	**Validation cohort (n=54)**	**t/χ^2 value**	***P* value**
Age, years, mean (SD)	62.5 ± 8.7	63.2 ± 8.1	0.521	0.603
Male, n (%)	74 (58.7)	30 (55.6)	0.172	0.678
BMI, kg/m^2, mean (SD)	23.30 (3.80)	23.15 (3.65)	0.245	0.807
Smokers, n (%)	75 (59.5)	32 (59.3)	0.001	0.971
Drinkers, n (%)	67 (53.2)	29 (53.7)	0.005	0.944
Family history of CRC, n (%)	25 (19.8)	10 (18.5)	0.049	0.825
FIT-positive, n (%)	79 (62.7)	34 (63.0)	0.002	0.968
HGB, g/L, mean (SD)	87.8 (26.9)	87.1 (27.6)	0.163	0.871
CEA, $\log_{10}$μg/mL, mean (SD)	2.41 (0.73)	2.47 (0.79)	0.487	0.627
CA19-9, $\log_{10}$IU/mL, mean (SD)	2.44 (0.68)	2.50 (0.74)	0.532	0.596
Tumor location, colonic, n, (%)	70 (55.6)	29 (53.7)	0.058	0.810
Tumor size, ≥5cm, n, (%)	66 (52.4)	28 (51.9)	0.005	0.945
TNM stage, n,（I/II/III/IV）	25/36/40/25	11/16/22/5	2.341	0.505

CRC：colorectal cancer；BMI：Body Mass Index；FIT：Fecal Immunochemical Test；HGB：Hemoglobin；CEA：Carcinoembryonic Antigen；CA19-9：Carbohydrate Antigen 19-9.

Supplementary Table 3. Clinical variables associated with multi-gene methylation positive in multivariate analysis

Variables	β value	Wald χ^2 value	OR	95% CI	*P* value
Age (≥60 years old vs. <60 years old)	0.365	4.591	1.264	1.265-2.358	0.369
Sex (female vs. male)	0.120	0.850	1.127	0.945-1.345	0.456
TNM stage (per stage)	0.750	9.320	2.117	1.452-3.087	0.002
Tumor size (≥ 5cm vs. < 5cm)	0.680	7.890	1.974	1.321-2.950	0.005
Tumor location (colonic vs. ileal)	-0.150	1.230	0.861	0.672-1.103	0.267
CEA (elevated vs. normal)	0.080	0.420	1.083	0.892-1.315	0.517
CA19-9 (elevated vs. normal)	0.050	0.180	1.051	0.876-1.261	0.671

CEA：Carcinoembryonic Antigen；CA19-9：Carbohydrate Antigen 19-9.

Supplementary Figure 1. Representative amplification curves for the methylated standards.

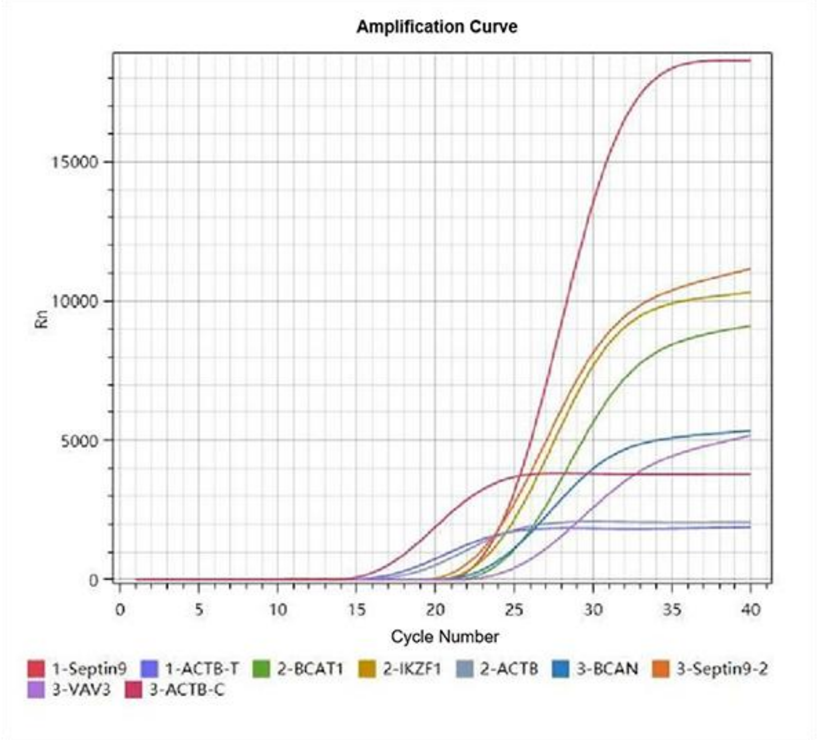

Supplement: Supplementary file 1 [file DataSheet1.pdf]
